# Editorial Department

**Published:** 1847-09

**Authors:** 


DOCT. FLINT. BATAVIA, August 14th, 1847.

DEAR SIR:—Permit me through the columns of the Medical Journal to express my deep and earnest gratitude to the Medical Faculty of your city, for their kind attentions and warm sympathies during the last illness of a beloved daughter. My grateful acknowledgments are especially due to Doctors Sprague and Cary.

Your obt. servant, J. A. BILLINGS, M. D.

